# Rearing Room Affects the Non-dominant Chicken Cecum Microbiota, While Diet Affects the Dominant Microbiota

**DOI:** 10.3389/fvets.2016.00016

**Published:** 2016-02-25

**Authors:** Jane Ludvigsen, Birger Svihus, Knut Rudi

**Affiliations:** ^1^Department of Chemistry, Biotechnology and Food Science, Norwegian University of Life Sciences, Ås, Norway; ^2^Department of Animal and Aquacultural Sciences, Norwegian University of Life Sciences, Ås, Norway

**Keywords:** chicken, microbiota, 16S rRNA gene, cecum, *Clostridium perfringens*

## Abstract

The combined effect of environment and diet in shaping the gut microbiota remains largely unknown. This knowledge, however, is important for animal welfare and safe food production. For these reasons, we determined the effect of experimental units on the chicken cecum microbiota for a full factorial experiment where we tested the combined effect of room, diet, and antimicrobial treatment. By Illumina Deep sequencing of the 16S rRNA gene, we found that diet mainly affected the dominant microbiota, while the room as a proxy for environment had major effects on the non-dominant microbiota (*p* = 0.006, Kruskal–Wallis test). We, therefore, propose that the dominant and non-dominant microbiotas are shaped by different experimental units. These findings have implications both for our general understanding of the host-associated microbiota and for setting up experiments related to specific targeting of pathogens.

## Introduction

The gut microbiota plays a crucial role for the host health through providing essential metabolites and vitamins, in addition to immune/gut maturation and protection toward pathogen colonization (1). Despite this crucial role, our knowledge about the ecological driving forces shaping the gut microbiota is limited. Although it is well known that diet and antimicrobial compounds can affect the gut microbiota, the influence of the environment is still largely unknown (2). This represents a major challenge when setting up experiments involving antimicrobial and/or dietary perturbations of the gut microbiota.

Here, we evaluated the effect of different experimental units in a full factorial experimental design, where both the microbiota composition and the level of *Clostridium perfringens* were determined for the chicken cecum microbiota. The experimental units evaluated were room as a proxy for ­environment, diet, and antimicrobial treatment. For the microbiota composition, we used Illumina deep sequencing of the 16S rRNA gene (3), while the level of *C. perfringens* was determined by real-time PCR (4).

The rationale for the choice of chicken microbiota and *C. perfringens* association is that chickens are kept in large flocks, with the potential for rapid, large-scale pathogen transmission (5). Since chickens do not have contact with the adult population other than from bacteria potentially ­colonizing the egg shell, they are prone to colonization by the environmental microbiota (6–8). *C. perfringens* represents a major challenge in poultry production (9). Traditionally, prophylactic use of antibiotics has been applied in *C. perfringens* control, but due to the spread of antibiotic resistance, prophylactic use of antibiotics is now banned or will be banned in most countries. Challenges related to banning antimicrobial compounds, however, are both the lack of alternatives for pathogen control and the lack of knowledge about the ecology of chicken gut microbiota (10).

## Materials and Methods

### Experimental Design

**Figure 1:**
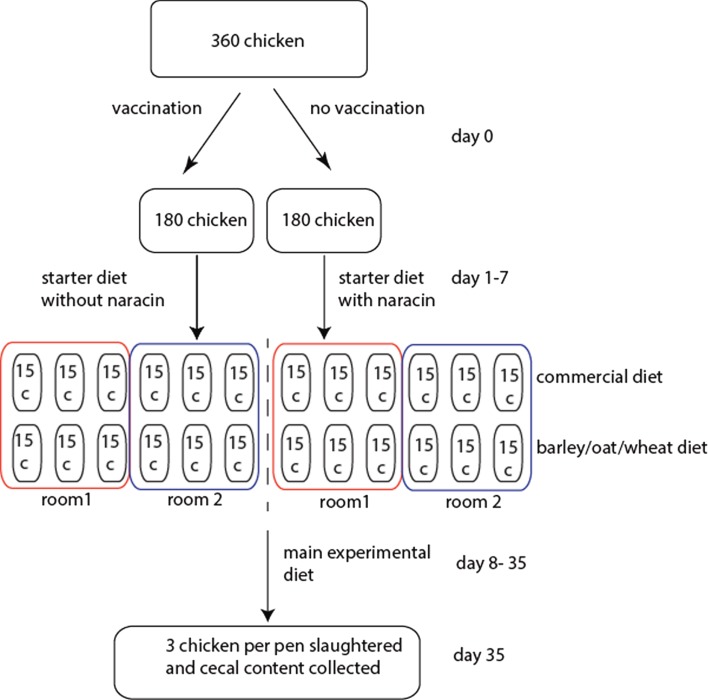
**Schematic outline of the experimental design**. For the rooms, the boxes represent pens with the respective number of chickens.

A total of 360 male broiler chickens of the breed Ross 308 were used in the experiment. Half of these were vaccinated against coccidiosis upon arrival by spraying Paracox-8 (Schering-Plough Ltd., UK) on the feed, and each of these two groups were then distributed among 12 pens (15 birds per pen) divided between two rooms (6 pens per room per treatment). The physical environment should be identical in the two rooms. All chickens received commercial starter diets until 7 days of age, where the non-vaccinated chickens were also given the antimicrobial narasin (Monteban, Elanco Animal Health, USA) throughout the experiment to prevent coccidiosis. Narasin, however, also have cross-inhibition toward *C. perfringens* (11). After 7 days, each of the treatment groups was split in two, and half of the pens continued to receive a commercial diet, while the other half received an equal portion of a barley/oats/wheat experimental diet. The rationale for the experimental diet was to utilize locally produced grains to reduce transportation costs. This resulted in four treatment groups with six pens per groups with three pens per room. After 4 days of adaptation to the new diets, at 11 days of age, birds were weighed in groups. Birds were also weighed prior to slaughter at 34 days of age. At 35 days of age, the chickens were slaughtered and the cecal contents from 3 birds per pen (72 birds in total) were collected for microbiota analyses. The birds were killed by cervical dislocation, and one randomly selected cecum was immediately dissected out from each animal. The experiments were conducted following Norwegian legislation and guidelines. The experimental design is schematically outlined in Figure 1.

### Microbiota Analyses

The complete content of one cecum for each chicken was suspended 1:3 in STAR buffer (Roche, Switzerland). The samples were then immediately frozen at −20°C, with further processing within 1 month.

Thawed samples were vortexed, and 500 μl of the liquid phase was transferred to tubes with acid-washed glass beads (Sigma-Aldrich, <106 μm; 0.25 g). Subsequently, the samples were processed twice in a MagNaLyzer (Roche, Switzerland) at 6500 rpm for 20 s with cooling using the MagNaLyzer cooler Wein-between to disrupt the cells. Cell debris was removed by centrifugation at 19,000 × *g* for 5 min. Subsequent DNA extraction was done using MagMiniLGC kit (LGCgenomics, UK), following the manufacturer’s recommendations using a KingFisher Flex (Thermo Scientific, USA) DNA extraction robot.

A nested approach was used for the 16S rRNA gene Illumina sequencing. The first PCR was run for 25 cycles using the primers and protocol developed by Yu et al. (12). The PCR product was then diluted 1:100, with subsequent 10 PCR cycles following the protocol by Naseribafrouei et al. (3) using Illumina MiSeq V3 kit (Illumina, USA). Resulting 300 bp paired-end data were analyzed using the QIIME pipeline (13). Sequences were paired-end joined (*fastq-join*) and quality filtered based on average sequence quality score more than 25. Then, sequences were clustered with 97% identity level using *usearch* v7 (14, 15). Taxonomic assignments were done using the Greengenes (16) and the RDP database (17).

For the categorical variables room, diet, and antimicrobial treatment, we used partial least square discriminant (PLS-DA) analyses for relating the variables with the operational taxonomic unit (OTU) table, while for *C. perfringens* we used partial least square (PLS) analyses. In all cases, we used Venetian Blinds cross validation and the average microbiota within each pen as explanatory variables. These analyses were done using the PLS toolbox (Eigenvector, USA), running in the Matlab environment (Mathworks Inc., USA).

We used Simpson’s *D* and observed species as alpha diversity measures whereas we used Bray–Curtis and Jaccard indexes for beta diversity analyses. The non-parametric Kruskal–Wallis test was used to test the significance of the differences detected for the diversity measures (Minitab Inc., USA).

For the quantitative PCR, we first quantified the 16S rRNA gene using the previously described PRK primers (12). For *C. perfringens* detection, we used real-time PCR targeting the toxin gene cpe (154 bp), as described previously by Rinttila et al. (4). Both the rtPCRs were run on a LightCycler 480 (Roche, Switzerland) with evagreen PCR chemistry (Solis BioDyne, Estonia). The chickens were scored positive for the respective toxin genes given that the PRK PCR gave a ct below 20 and toxin gene PCR below <35, while the corresponding negative assignments were the cases where PRK PCR gave a ct below 20 and the toxin gene PCR gave a ct of 35, or above. The rationale for the qualitative assignments was the expected low quantitative levels of *C. perfringens*. ANOVA (Minitab) was used to analyze the relationship between the qPCR data, room, diet, and antimicrobial treatment.

## Results

### Growth Characteristics and Mortality

Analysis of variance showed no significant effects on weight gain, with an average weight gain of 2.2 kg from day 11 to 34. A large numerical difference in mortality was observed during the first 11 days of life. The chickens that did not receive narasin showed a mortality of 11%, while the chickens that received narasin showed a mortality of 0.5%. From 11 to 34 days of age, the numerical difference decreased: 1.8% for the narasin group and 4.4% for the non-narasin group. As similar difference was observed for the experimental diet as compared to the commercial (average 4.7 vs. 1.8%, respectively) diet. Room did not appear to have any appreciable effect on mortality.

### Microbiota Composition

A total of 4.7 Gbp 16S rRNA gene sequence data with 84% above Q30 was generated by Illumina sequencing. After assembly and quality filtering, average number of sequences per sample was 49,037, with only 1 out of 72 samples with <4000 sequences. We therefore rarefied the samples to 4000 sequences prior to further analysis.

Analyses of the taxonomic assigned data showed that the overall microbiota composition was dominated by *Firmicutes* at the phylum level, *Clostridia* at the class level, while at the order level most of the sequences were unclassified, suggesting a high level of poorly characterized bacteria (Figure S1 in Supplementary Material).

**Figure 2:**
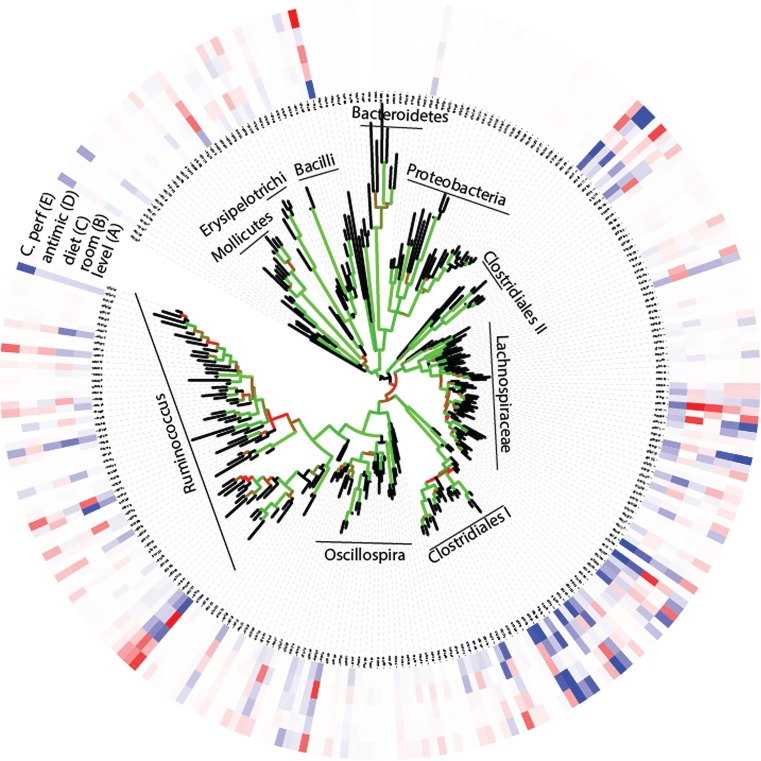
**OTU associations (A) with relative composition, (B–D) experimental factors, and (E) *C. perfringens***. (A) The inner circle represents the relative composition in percentage, while the other circles (B–E) represent loadings (OTU importance) in the respective regression models. The color code for the circles are given by that blue represents high (greater than 3) and red low (less than −3) values. The tree shows the phylogenetic association of the OTUs, with the Bootstrap support being given by the color code of the branches (black <60%, red 60–80%, and green >80%).

Due to the high number of taxonomically unassigned sequences, we pursued our further analyses at the OTU level using the phylogenetic tree as a proxy for taxonomy. These analyses revealed an overall large observed microbial species richness (*n* = 273), with only a few dominant OTUs (Figure 2). For the whole tree, there were two lineages that could not be taxonomically assigned beyond the class level. These were denoted Clostridiales I and II, respectively (Figure 2). Comparison of the relative distribution of OTUs with that of the expected log normal distribution confirmed an overrepresentation for seven OTUs with an average abundance >3% (Figure S2 in Supplementary Material). All the seven overrepresented OTUs have previously been identified in poultry, but they generally lack closely related taxonomically assigned sequences (Table S1 in Supplementary Material).

### Effect of Room, Feed, Antimicrobial Treatment, and Pen on the Microbiota Composition

We first evaluated the effect of pen by comparing the variance for each OTU between the three birds per pen with the variance between the three pens within the treatment groups. For 76% of the OTUs, the variance was lower between the pens within the same treatment group than among birds in the same pen.

Using PLS-DA, we obtained an overall significant association between the experimental factors and microbiota, with a respective cross-validated accuracy of classification for room, diet, and antimicrobial treatment of 0.70, 0.78, and 0.60. This means that the microbiota composition can be predicted based on the experimental factors. There were no internal correlations between the OTUs important for these associations (*p* > 0.1, Spearman correlation), with a relatively complex pattern in which closely related OTUs have opposite influences in the classification models (Figure 2). This means that closely related bacteria can have opposite relationships to the factors investigated.

**Figure 3:**
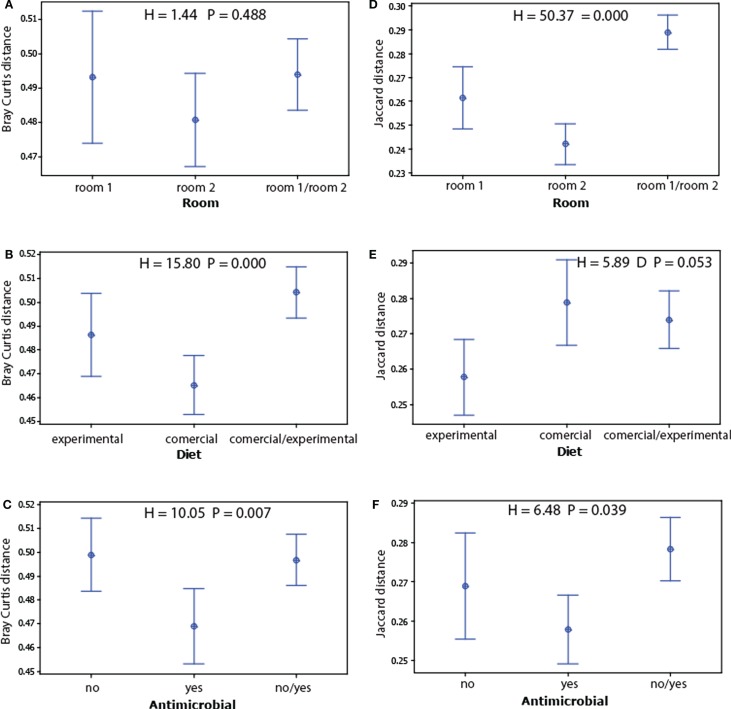
**Main beta diversity associations of the microbiota with the experimental factors**. The beta diversity within and between the factors levels for each treatment were determined for Bray–Curtis **(A–C)** and Jaccard distances **(D–F)**. The dots represent mean values, while the error bars represent the 95% confidence intervals. The overall significance for each model was tested by Kruskal–Wallis test.

We found no significant associations for alpha diversity (Simpson’s *D* and observed species), whereas for beta diversity, we found strong associations for room and the Jaccard index (presence absence of OTU’s), the Bray–Curtis index to diet (taking into account OTU levels) showed the strongest association (Figure 3).

**Figure 4:**
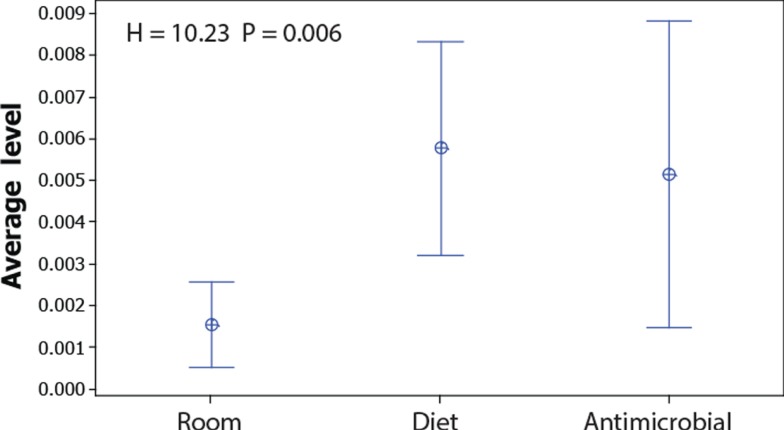
**Abundance of OTUs significantly associated with room, diet, and antimicrobial treatment**. The dots represent the mean values, while the error bars represent the 95% confidence interval for the OTUs showing significant false discovery corrected associations with the respective factors (raw data presented in Table S2 in Supplementary Material). The overall significance was tested by Kruskal–Wallis test.

Since the non-dominant microbiota have a larger impact on the Jaccard index than the dominant, and because the Bray–Curtis index is mostly influenced by dominant OTUs, we compared the levels of the OTUs that show significant false discovery corrected associations with the experimental factors (Table S2 in Supplementary Material). These comparisons showed that the average level of the OTUs associated with room differences was significantly lower than for those associated with diet (Figure 4).

### Associations to *C. perfringens*

Using *plc* as a proxy for *C. perfingens*, we established a significant association with antimicrobial treatment, where narasin showed a major reduction in *C. perfringens* prevalence (63 vs. 17%, *p* = 0.001 ANOVA test for non-treated and narasin-treated chickens, respectively). The effect was independent of room.

The direct correlation between *C. perfringens* and the microbiota was investigated by PLS regression. A four component model showed the best correlation (*R*^2^ = 0.94 for calibration and 0.04 for validation). Mapping the loadings onto the OTU-derived phylogenetic tree shows OTUs with both positive and negative *C. perfringens* associations across the tree (Figure 2). OTU 20 (within the Clostridiales I cluster in Figure 2) showed the largest influence on the model (loading = 23.3). This OTU also show a significant direct positive correlation with *C. perfringens* (*p* = 0.002, Spearman correlation). However, there were no single OTUs with pronounced negative associations to *C. perfringens*.

## Discussion

Our main finding was the presence of a diverse but low-abundant microbiota associated with the experimental unit room, while diet mainly affected the high-abundant microbiota. Since the physical environment is designed to be similar in the two rooms, the differences detected could potentially be due to differences in the room microbiota, and as a consequence a difference in microbial exposure (18). A likely source for the potential room associated microbiota is clostridial spores, since these can easily survive decontamination (19).

The high abundant microbiota – being more abundant in the population than expected from a lognormal distribution – seemed host specific (OTUs were poultry associated as determined by Blast searches). The distinct distribution patterns of high- and low-abundant species resemble a common pattern in many ecosystems (20). Related distributions have also been observed for the human gut microbiota, with a common core of bacteria shared among most individuals (21, 22). A host-specific microbiota has also been identified from cataloging mouse microbiota (23). Taken together, our results contribute to the support of a model advocating the importance of a host-specific microbiota (24).

Several closely related OTUs show opposite relationship to the experimental factors investigated. This highlights the importance of high-resolution analyses of the microbiota. Furthermore, we identified two abundant clusters of OTUs denoted Clostridiales I and II with no closely related counterparts in the databases. The dominance of *Clostridium* spp. in the chicken cecum has previously been noted in several studies (6–8). However, despite the importance of clostridia in the chicken gut, this class is poorly characterized taxonomically (25). This renders the risk of overseeing or misinterpreting effects when using taxonomic model-based approaches.

Since there are no direct transmission routes of bacteria from mother to offspring for chickens other than potentially through the egg shell, most of the host-associated microbiota is probably transmitted at a later stage than egg laying. *Clostridia* are generally spore-forming and widely transmitted at the farm level (26). Thus, spores could be the main vector for host-specific clostridia colonization for chickens. The importance of spores in establishing a host-specific microbiota has also recently been noted for humans (27, 28).

The antimicrobial effect of narasin on *C. perfringens* seems independent of the effect on the cecum microbiota, because the OTUs correlating with narasin treatment are not the same as those correlating with *C. perfringens*. This may be due to the fact that the main reservoir of *C. perfringens* is in the small intestine, with the main effect of narasin being in the small intestine because of the mucus association of bacteria. Furthermore, the effect of narasin could also potentially be indirect There were, however, OTUs that correlated directly both positively and negatively with *C. perfringens* in the cecum independent of the narasin treatment. In the cecum, most of the bacteria are lumen associated, which may explain the different interaction pattern here. The strongest positive correlation was detected for an OTU within the uncharacterized Clostridiales I group. Since no information is available for these clostridia, we cannot deduce potential mechanisms for the correlations, or whether intervention strategies through the microbiota would be feasible.

The main conclusion from our work is that the experimental units affect the dominant and non-dominant microbiota differently. These differences need to be considered when investigating the effect of dietary and antimicrobial interventions.

## Author Contributions

JL conducted the analytical work, BS designed and carried out the chicken experiments, and KR wrote the paper and analyzed the data.

## Conflict of Interest Statement

The authors declare that the research was conducted in the absence of any commercial or financial relationships that could be construed as a potential conflict of interest. Nortura is a meat producing company. None of the authors are associated with Nortura, and there were no restrictions on data reporting.
