# Supplementary material for: Rearing Room Affects the Non-dominant Chicken Cecum Microbiota, While Diet Affects the Dominant Microbiota
Source: Front Vet Sci. 2016 Feb 25;3:16. doi: 10.3389/fvets.2016.00016 (PMC4766280; doi:10.3389/fvets.2016.00016)
Supplement: Supplementary file 1 [file data_sheet_1.pdf]

SUPPLEMENTARY TABLES

Supplementary Table 1. Relations of the most abundant OTU's

Query	Abundance (%)	Origin[1]	Taxonomy[3]	Identity (%)
OTU_1	3.2	chicken	Ruminococcus torques strain VPI B2-51	95.7
OTU_2	3.2	turkey/human IBD[2]	Anaerobacterium chartisolvens strain T-1-35	86.0
OTU_5	4.3	turkey/human IBD[2]	Anaerobacterium chartisolvens strain T-1-35	84.6
OTU_6	4	chicken/human IBD[2]	Lactobacillus kitasatonis strain JCM 1039	92.5
OTU_20	3.5	turkey/human IBD[2]	Vallitalea pronyensis strain FatNI3	87.0
OTU_22	3.5	chicken	Clostridium glycyrrhizinilyticum strain ZM35	94.3
OTU_139	4.7	turkey	Vallitalea pronyensis strain FatNI3	87.3

[1] Host microbiomes with sequences > 97% identity for non-taxonomical assigned sequences

[2] Mucosal Microbiome from Indian Patients (unpublished)

[3] Closest taxonomically assigned match in NCBI

Supplementary Table 2. Associations between OTU levels and experimental factors

OTU	Average level[1]	Room[2]	Diet[2]	Antimicrobial[2]
OTU_68	0.003	0	0	1
OTU_199	0.016	0	0	1
OTU_8	0.031	0	1	0
OTU_139	0.032	0	0	0
OTU_46	0.010	0	1	0
OTU_35	0.018	0	0	0
OTU_120	0.006	0	0	0
OTU_5	0.032	0	0	0
OTU_4	0.025	0	0	1
OTU_100	0.005	0	0	0
OTU_22	0.043	0	0	0
OTU_79	0.001	0	0	0
OTU_173	0.004	0	1	0
OTU_278	0.027	0	0	0
OTU_10	0.015	1	0	0
OTU_28	0.012	0	1	0
OTU_12	0.011	0	0	0
OTU_189	0.000	0	1	0
OTU_14	0.012	0	1	0
OTU_56	0.002	0	0	1
OTU_16	0.014	0	1	0
OTU_106	0.006	0	0	0
OTU_48	0.004	0	0	0
OTU_39	0.003	0	0	0
OTU_62	0.004	0	0	1
OTU_26	0.013	0	0	0
OTU_20	0.040	0	0	1
OTU_91	0.003	0	0	0
OTU_58	0.002	0	1	0
OTU_99	0.029	0	0	0
OTU_98	0.015	0	0	0
OTU_235	0.001	0	0	0

OTU_109	0.002	0	1	0
OTU_260	0.010	0	0	0
OTU_34	0.012	0	0	0
OTU_85	0.016	0	0	0
OTU_19	0.012	0	0	0
OTU_27	0.006	0	0	0
OTU_6	0.035	0	1	0
OTU_18	0.010	0	1	0
OTU_135	0.001	0	0	0
OTU_73	0.003	0	1	0
OTU_44	0.007	1	0	0
OTU_42	0.007	0	0	0
OTU_45	0.002	0	0	0
OTU_47	0.006	0	0	0
OTU_187	0.002	0	0	0
OTU_31	0.007	0	0	0
OTU_143	0.000	0	1	0
OTU_83	0.001	0	0	1
OTU_234	0.006	0	0	0
OTU_41	0.005	0	1	0
OTU_121	0.000	0	0	0
OTU_59	0.009	0	0	0
OTU_130	0.000	0	0	0
OTU_114	0.001	0	0	0
OTU_2	0.035	0	0	0
OTU_3	0.008	0	1	0
OTU_97	0.001	1	0	0
OTU_1	0.047	0	0	0
OTU_64	0.003	0	0	1
OTU_104	0.015	0	0	0
OTU_93	0.001	1	0	0
OTU_57	0.002	0	0	0
OTU_29	0.008	0	0	0
OTU_38	0.003	1	1	0
OTU_77	0.001	0	0	1

OTU_154	0.000	1	0	0
OTU_112	0.009	0	0	0
OTU_123	0.001	0	0	0
OTU_15	0.006	0	0	1
OTU_23	0.010	0	0	0
OTU_90	0.001	0	1	0
OTU_105	0.007	0	1	0
OTU_126	0.000	1	0	0
OTU_181	0.000	0	0	1
OTU_43	0.003	0	1	0
OTU_9	0.005	0	0	0
OTU_113	0.003	0	0	0
OTU_127	0.016	0	1	1
OTU_193	0.000	1	0	0
OTU_49	0.007	0	1	0
OTU_72	0.003	0	0	0
OTU_55	0.003	0	0	0
OTU_65	0.003	0	1	0
OTU_168	0.001	0	0	1
OTU_108	0.020	0	1	0
OTU_33	0.002	0	0	0
OTU_115	0.001	1	0	0
OTU_158	0.000	0	0	1
OTU_70	0.004	1	0	0
OTU_50	0.007	0	0	0
OTU_7	0.009	0	0	0
OTU_107	0.001	1	0	0
OTU_30	0.011	0	0	1
OTU_101	0.002	0	0	0
OTU_96	0.001	0	0	0
OTU_148	0.001	0	0	0
OTU_54	0.002	0	0	0
OTU_116	0.004	0	1	0
OTU_133	0.002	0	0	0
OTU_74	0.002	1	0	0

OTU_132	0.000	0	0	0
OTU_195	0.000	1	0	0
OTU_267	0.000	0	0	0
OTU_165	0.000	0	0	0
OTU_87	0.000	0	1	0
OTU_151	0.001	0	1	0
OTU_119	0.001	0	0	0
OTU_13	0.008	0	1	0
OTU_124	0.005	0	0	0
OTU_92	0.001	0	0	0
OTU_88	0.001	0	0	0
OTU_67	0.001	0	0	0
OTU_174	0.001	1	0	0
OTU_140	0.000	0	0	0
OTU_66	0.003	0	0	0
OTU_89	0.002	0	0	0
OTU_153	0.000	0	0	0
OTU_81	0.000	0	0	0
OTU_202	0.001	0	0	0
OTU_25	0.005	0	0	0
OTU_170	0.000	0	0	0
OTU_175	0.000	0	0	0
OTU_141	0.000	0	1	0
OTU_270	0.000	0	0	0
OTU_233	0.000	0	0	0
OTU_95	0.002	0	0	0
OTU_71	0.002	0	0	0
OTU_84	0.001	0	1	0
OTU_216	0.000	0	0	0
OTU_264	0.000	0	0	0
OTU_171	0.002	0	0	0
OTU_167	0.000	0	0	0
OTU_61	0.002	0	0	1
OTU_129	0.001	0	1	1
OTU_166	0.000	1	0	0

OTU_76	0.002	0	0	1
OTU_37	0.004	0	0	0
OTU_86	0.001	1	0	0
OTU_52	0.001	0	0	0
OTU_80	0.001	0	1	0
OTU_53	0.004	0	0	0
OTU_157	0.000	0	0	1
OTU_273	0.000	0	0	0
OTU_276	0.013	0	0	0
OTU_146	0.000	1	0	0
OTU_208	0.000	0	0	0
OTU_229	0.000	1	0	1
OTU_223	0.000	0	0	0
OTU_161	0.000	0	0	0
OTU_136	0.000	0	0	1
OTU_63	0.003	0	0	0
OTU_11	0.007	1	1	0
OTU_196	0.000	0	0	0
OTU_203	0.000	0	1	0
OTU_155	0.000	1	0	0
OTU_131	0.000	0	0	0
OTU_182	0.004	0	0	0
OTU_184	0.000	0	0	0
OTU_145	0.000	0	0	0
OTU_134	0.000	0	1	0
OTU_169	0.000	0	0	0
OTU_122	0.000	0	0	0
OTU_206	0.000	1	0	0
OTU_103	0.001	0	0	0
OTU_160	0.000	0	0	0
OTU_192	0.000	0	0	1
OTU_138	0.000	1	0	0
OTU_228	0.000	0	0	0
OTU_137	0.001	0	1	0
OTU_60	0.003	0	0	0

OTU_40	0.002	0	1	1
OTU_188	0.000	0	0	0
OTU_265	0.000	1	0	0
OTU_198	0.000	0	0	0
OTU_209	0.000	0	0	0
OTU_215	0.000	0	0	0
OTU_111	0.001	0	0	0
OTU_205	0.000	1	0	0
OTU_17	0.013	0	0	0
OTU_51	0.002	0	0	0
OTU_200	0.000	0	0	0
OTU_204	0.000	0	0	0
OTU_242	0.000	0	0	0
OTU_217	0.000	0	0	0
OTU_222	0.000	0	0	0
OTU_232	0.000	0	0	0
OTU_163	0.000	0	0	0
OTU_246	0.000	0	0	0
OTU_258	0.000	0	0	0
OTU_255	0.000	1	0	0
OTU_211	0.000	0	0	0
OTU_156	0.000	0	0	0
OTU_177	0.000	0	1	0
OTU_144	0.000	0	0	0
OTU_279	0.000	0	0	0
OTU_179	0.000	0	0	0
OTU_207	0.000	0	0	0
OTU_69	0.003	1	0	0
OTU_220	0.000	0	0	0
OTU_259	0.000	0	0	0
OTU_152	0.000	0	1	0
OTU_147	0.000	0	0	0
OTU_213	0.000	0	0	0
OTU_21	0.007	0	0	0
OTU_185	0.000	1	0	0

OTU_218	0.000	0	0	0
OTU_75	0.001	0	0	1
OTU_110	0.000	0	0	0
OTU_250	0.000	0	0	0
OTU_248	0.000	0	0	0
OTU_257	0.000	0	0	0
OTU_268	0.000	0	0	0
OTU_24	0.001	0	0	0
OTU_201	0.000	0	0	0
OTU_237	0.000	0	0	0
OTU_238	0.000	0	0	0
OTU_214	0.000	0	0	0
OTU_186	0.000	0	0	0
OTU_261	0.000	0	1	0
OTU_159	0.000	1	0	0
OTU_178	0.000	0	0	0
OTU_256	0.000	0	0	0
OTU_36	0.001	1	0	0
OTU_32	0.002	0	0	0
OTU_82	0.000	0	0	0
OTU_125	0.002	0	0	0
OTU_94	0.001	1	0	0
OTU_191	0.000	0	0	0
OTU_180	0.000	0	0	0
OTU_118	0.000	1	0	0
OTU_78	0.000	0	0	0
OTU_245	0.000	1	0	0
OTU_102	0.001	1	0	0
OTU_197	0.000	0	1	1
OTU_230	0.000	0	0	0
OTU_263	0.000	0	0	0
OTU_212	0.000	0	0	0
OTU_176	0.000	0	0	1
OTU_262	0.000	0	0	0
OTU_275	0.000	0	0	0

OTU_219	0.000	0	0	1
OTU_225	0.000	0	0	0
OTU_252	0.000	0	0	0
OTU_150	0.000	0	0	0
OTU_271	0.000	0	0	0
OTU_226	0.000	0	0	0
OTU_253	0.000	0	0	0
OTU_227	0.000	0	0	0
OTU_244	0.000	0	0	0
OTU_231	0.000	0	0	0
OTU_149	0.000	0	0	0
OTU_117	0.000	1	0	0
OTU_239	0.000	0	0	0
OTU_241	0.000	0	0	0
OTU_224	0.000	0	0	0
OTU_249	0.000	0	0	0
OTU_274	0.000	0	0	0
OTU_190	0.000	0	0	0
OTU_162	0.000	0	1	0
OTU_277	0.000	0	0	0
OTU_221	0.000	0	0	0
OTU_183	0.000	0	0	0
OTU_272	0.000	0	0	0
OTU_128	0.000	0	0	0
OTU_194	0.000	0	0	0
OTU_142	0.000	0	0	0
OTU_266	0.000	0	0	0
OTU_210	0.000	0	0	0
OTU_164	0.000	0	0	0
OTU_172	0.000	0	0	0
OTU_240	0.000	0	0	0

[1] Represents the average level for all samples [2] 1 – false discovery corrected p values < 0.05 and; 0 – false discovery corrected p values > 0.05

SUPPLEMENTARY FIGURES

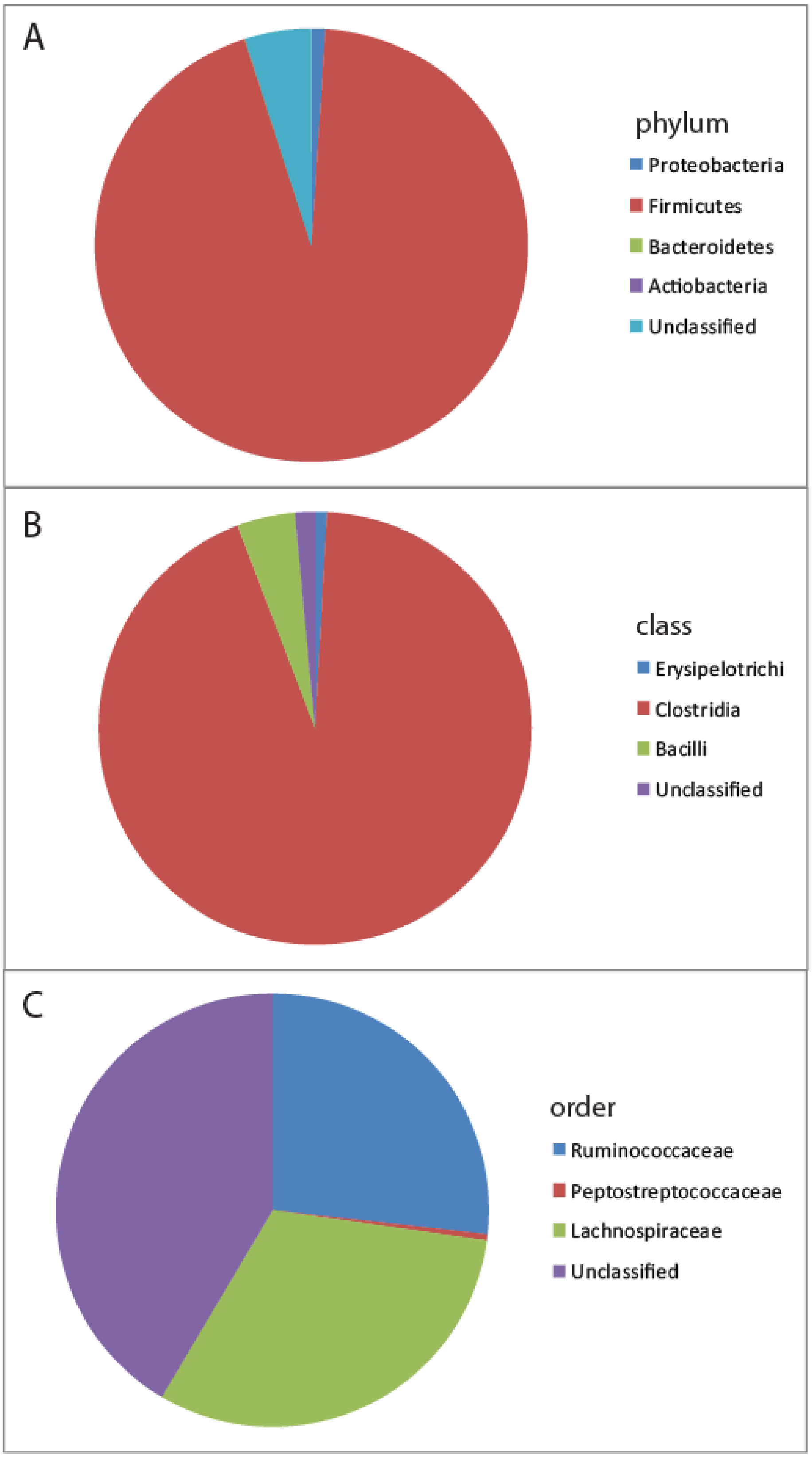

Supplementary Figure 1. Overall taxonomic composition of cercal microbiota as determined by the RDP hierarchical classifier. (A) phylum level, (B) class level and (C) order level. The taxonomic levels are based on RDP taxonomy.

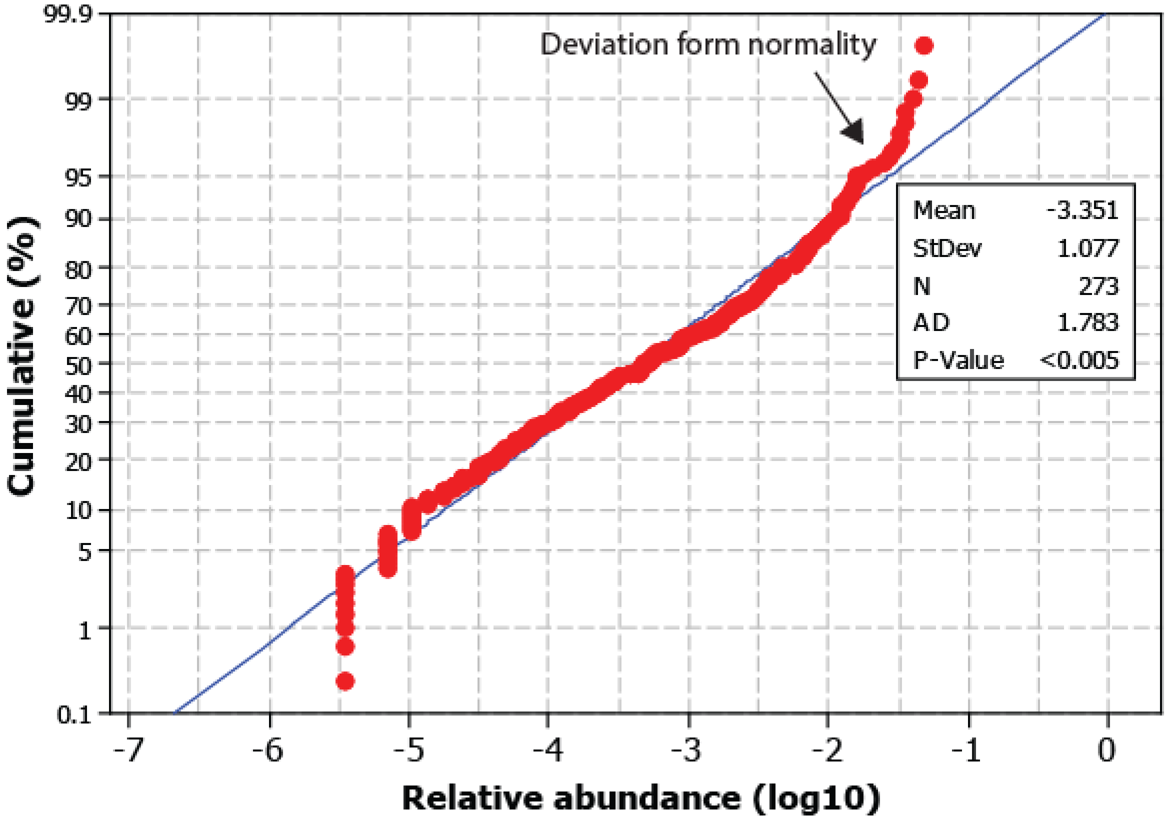

Supplementary Figure 2. Probability plot for average log abundance. The plot illustrates the expected distribution given normality. The Arrow indicate the seven OTU's with higher abundance than expected from a normal distribution.
